# Different Mode of Afferents Determines the Frequency Range of High Frequency Activities in the Human Brain: Direct Electrocorticographic Comparison between Peripheral Nerve and Direct Cortical Stimulation

**DOI:** 10.1371/journal.pone.0130461

**Published:** 2015-06-18

**Authors:** Katsuya Kobayashi, Riki Matsumoto, Masao Matsuhashi, Kiyohide Usami, Akihiro Shimotake, Takeharu Kunieda, Takayuki Kikuchi, Nobuhiro Mikuni, Susumu Miyamoto, Hidenao Fukuyama, Ryosuke Takahashi, Akio Ikeda

**Affiliations:** 1 Department of Neurology, Kyoto University Graduate School of Medicine, Kyoto, Japan; 2 Department of Epilepsy, Movement Disorders and Physiology, Kyoto University Graduate School of Medicine, Kyoto, Japan; 3 Human Brain Research Center, Kyoto University Graduate School of Medicine, Kyoto, Japan; 4 Department of Neurosurgery, Kyoto University Graduate School of Medicine, Kyoto, Japan; 5 Department of Neurosurgery, Sapporo Medical University School of Medicine, Sapporo, Hokkaido, Japan; Medical Photonics Research Center. Hamamatsu University School of Medicine, JAPAN

## Abstract

Physiological high frequency activities (HFA) are related to various brain functions. Factors, however, regulating its frequency have not been well elucidated in humans. To validate the hypothesis that different propagation modes (thalamo-cortical vs. cortico-coritcal projections), or different terminal layers (layer IV vs. layer II/III) affect its frequency, we, in the primary somatosensory cortex (SI), compared HFAs induced by median nerve stimulation with those induced by electrical stimulation of the cortex connecting to SI. We employed 6 patients who underwent chronic subdural electrode implantation for presurgical evaluation. We evaluated the HFA power values in reference to the baseline overriding N20 (earliest cortical response) and N80 (late response) of somatosensory evoked potentials (HFA_SEP(N20)_ and HFA_SEP(N80)_) and compared those overriding N1 and N2 (first and second responses) of cortico-cortical evoked potentials (HFA_CCEP(N1)_ and HFA_CCEP(N2)_). HFA_SEP(N20)_ showed the power peak in the frequency above 200 Hz, while HFA_CCEP(N1)_ had its power peak in the frequency below 200 Hz. Different propagation modes and/or different terminal layers seemed to determine HFA frequency. Since HFA_CCEP(N1)_ and HFA induced during various brain functions share a similar broadband profile of the power spectrum, cortico-coritcal horizontal propagation seems to represent common mode of neural transmission for processing these functions.

## Introduction

In the last decade, advancement in technology has made it possible to analyze ultra slow or high frequency activities (HFAs), or high frequency oscillations (HFOs) in the human brain. Neuronal functional networks show the broadband oscillatory activities ranging from 0.01 Hz to 600 Hz or above. In particular, neuronal activities above the conventional gamma range are reported to be specific to the each content of neuronal functional activities in both physiological and pathological neuronal processes [1–3].

Physiologically, gamma oscillations, in particular, those above 60 Hz (60–200 Hz) are closely associated with brain functions such as motor [1,4–6], language [7–8], attention [9], auditory [10–11], and visual [12] functions. Neuronal activities of the conventional ‘narrowband’ gamma (below 60–80 Hz) have been reported to originate from neuronal synchronization mainly by inhibitory interneuron networks [13–15], and those of ‘broadband’ high gamma (80–200 Hz) have been reported to reflect increase of the multiunit activities or local field potentials [16–18].

Much before the discovery of high gamma activities related with various brain functions, faster activity around 600 Hz was discovered and intensively investigated in somatosensory function by recording somatosensory evoked potentials (SEPs) [19–29]. This activity was different from the aforementioned activities in that it is an evoked activity that overrides the early cortical component (N20) of median nerve SEP. The generator of HFOs or HFAs of SEP (HFA_SEP_) has been proposed at various locations: the terminal segments of the thalamocortical fibers [30], the primary somatosensory cortex (SI) close to the generator of SEP N20 [31], GABAergic inhibitory fast-spiking interneurons at SI [26], and subcortical neurons [32]. Among them, the main generator has been proposed at or around SI from findings obtained by direct electrocorticographic recording. HFA_SEP_ seems to play an important role in sensory information processing, and their impairment is reported in patients with multiple sclerosis, migraine and epilepsy [33–36].

The pathological high frequency oscillations (HFOs) or HFAs have been recently explored in the field of epilepsy, and nowadays are regarded as one of the possible biomarkers of epileptogenicity [37–39]. Pathological HFAs are generally divided into ripples between 100 and 200 Hz and fast ripples more than 250 Hz [2]. The generator of pathological HFAs, especially fast ripples, is presumably single or recurrent population spikes that reflect summated hypersynchronized discharges of principal cells [40–41].

Besides intrinsic physiological or pathological HFAs, external stimulation can generate cortical HFAs. In addition to the peripheral nerve stimulation that generates HFA_SEP_, direct electrical cortical stimulation can produce HFOs. Electrical stimulation of the piriform cortex produced oscillatory gamma responses (50–60 Hz) mainly at the layer Ia (superficial layer) in normal rats [42]. Moreover a recent study has shown that pathological HFAs could reliably be produced by electrical microstimulation of the hippocampus in the tetanus toxin-induced epileptic rats and normal rats [43].

Although the mechanisms how HFAs are generated have been studied both *in vivo and vitro* in animals [44–45], factors regulating the frequency of physiological (60–200 Hz for cognitive HFA, ~600 Hz for HFA_SEP_) and pathological (epileptic: 80–500 Hz) HFAs have not been well elucidated in humans. We focused on the SI cortex to investigate factors regulating physiological HFAs since HFA_SEP_ (~600 Hz) has been extensively studied in humans. We hypothesized that different modes of propagation, vertical (thalamo-cortical) vs. horizontal (cortico-cortical) projections, or different terminal layers, i.e., layer IV vs. layer II/III affect the frequency of physiological HFAs. We compared HFAs in SI triggered by median nerve stimulation with those elicited by single pulse electrical cortical stimulation (SPES). Cortico-cortical evoked potentials (CCEPs) have been widely used to evaluate the cortical evoked response to SPES [46]. CCEPs are recorded from the adjacent and remote cortices by averaging electrocorticograms (ECoGs) time-locked to the single pulse stimuli. CCEPs usually consist of an early negative component (N1) and a late negative component (N2). This method has been used for investigating cortico-cortical connections involved in functional brain systems [46–54] or seizure networks [55–56]. In the present study, by applying SPES to the cortex connecting to SI, we focused on the HFAs overriding CCEP N1 and N2 components in the SI cortex and compared their feature with HFA_SEP_. In the present study, we adopted the terminology of “HFAs” for all the high frequency activities although “HFOs” have been used in the previous studies of the high frequency activities overriding SEPs. This is because we do not only focus on the ‘narrowband’ high frequency activities showing discrete oscillatory activities but also the ‘broadband’ activities.

## Materials and Methods

### Patients

Six patients (2 female), 4 with medically intractable partial epilepsy and 2 with brain tumor were studied (Table 1). All underwent chronic subdural electrode implantation covering the peri-rolandic area for the presurgical evaluation. In all but patient 5, the epileptic foci were away from the peri-rolandic area. In patient 5, although the lesion (brain tumor) was in the peri-rolandic area, the hand SI was located outside the lesion. Because of the ill-defined ictal onset in the scalp EEG and normal MRI findings, patient 1 underwent electrode implantation twice: the first implantation in the bilateral hemispheres to lateralize the seizure onset, and the second one in the right hemisphere to localize the epileptic focus. Therefore we measured SEPs and CCEPs in 7 hemispheres from 6 patient, i.e., 2 hemispheres from patient 1—patient 1L and patient 1R. Neurological examination was normal except patient 5 who showed slight paresis in the right lower extremity. The implanted electrodes were made of platinum with a recording diameter of 2.3 mm and a center-to-center interelectrode distance of 1 cm (Ad-Tech, Rachine, WI, USA) or with a recording diameter of 3 mm and a center-to-center interelectrode distance of 1 cm (Unique Medical Co., Ltd., Tokyo, Japan). As a part of the clinical presurgical evaluation, high frequency (50 Hz) electrical stimulation was performed for functional cortical mapping. Cortical mapping of the peri-rolandic area was performed in patients 3–6. To define the exact location of each electrode on the brain, subdural electrodes were co-registered to three dimensional volume-rendered MRIs, which were reconstructed from MPRAGE taken while electrodes were in place. The location of each electrode was identified on the 2D-MRI by using its signal void due to the property of the platinum alloy. The methodological details have been described elsewhere [46,57]. The central sulcus (CS) and its relationship to the electrodes were also identified by anatomical landmarks on the 3D-MRI.

**Table 1:** Patient profile.

Patient	1		2	3	4	5	6
Age, gender	23F		24M	29M	34M	40M	28F
Handedness	R		R	L	L	R	R
Epilepsy	FLE		FLE	TLE	Parieto-temporal Lobe Epilepsy	Peri-rolandic Epilepsy	PLE
Etiology	FCD type IA		FCD type IB	FCD type IA and HS	Posttraumatic injury and ischemic change (parietal) and HS and dysplastic change (temporal)	Oligoastrocytoma	DNT
Neurological Examination	Normal		Normal	Normal	Normal	Slight right lower extremity weakness	Normal
Recording hemisphere	L	R	L	L	R	L	R

FLE = frontal lobe epilepsy, TLE = temporal lobe epilepsy, PLE = parietal lobe epilepsy, FCD = focal cortical dysplasia, HS = hippocampal sclerosis, DNT = dysembryoplastic neuroepithelial tumor

The present study was approved by the Ethics Committee of Kyoto University Graduate School of Medicine (No. 443). Written informed consent was obtained from all patients.

### Data Acquisition of SEPs

Electrocorticograms (ECoGs) were recorded with a bandpass filter of 0.016–600 Hz and a sampling rate of 2,000 Hz in all patients (EEG-1100, Nihon Kohden, Tokyo, Japan) and analyzed off-line using Matlab software (Matlab version 7.12.0; the MathWorks Inc., MA). Cortical recordings from subdural electrodes were referenced to a scalp electrode placed on the skin over the mastoid process contralateral (patient 1R and 2–6) or ipsilateral (patient 1L) to the side of electrode implantation.

The median nerve contralateral to the side of electrode implantation was stimulated at the wrist (a square wave pulse of 0.3 ms duration at 0.3 Hz) (Electrical Stimulator SEN-7203, Nihon Kohden, Tokyo, Japan). The stimulus intensity was adjusted to 20% above the motor threshold. In all the patiens, SEPs were recorded before tapering the antiepileptic drugs in the first week of chronic electrode implantation. During recording, the patients were lying on the bed and requested not to perform any specific task under awake condition. At least 2 trials of 150 sweeps were averaged to confirm the reproducibility of responses. SEPs were obtained by off-line averaging ECoGs time-locked to the stimulus onset with a time window of 1,000 ms (from 300 ms before to 700 ms after the stimulus onset). The baseline was set for the first 200 ms: from 300 ms to 100 ms before the stimulus onset.

### Data Acquisition of CCEPs

The methodological details of CCEPs have been described elsewhere [46,49]. In brief, electrical stimulation was applied in a bipolar manner to a pair of adjacently placed subdural electrodes by a constant-current stimulator (MEE-1232, Nihon Kohden, Tokyo, Japan). The single pulse electrical stimuli (a square wave pulse of 0.3 ms duration) were delivered in alternating polarity at a fixed frequency of 1 Hz. In the previous CCEP study, the CCEP consisted of an early (N1) and a late (N2) negative potentials, and the latencies of N1 and N2 usually ranged 10–50 ms and 100–200 ms, respectively. The stimulus intensity was set at 6–12 mA after confirming the absence of afterdischarges (ADs) and excessive artifacts that obscured the CCEP waveform.

ECoGs were recorded with a bandpass filter of 0.08–600 Hz. The sampling rate and reference electrode setting were the same as those for SEPs. During the recording of CCEPs, the patients were lying on the bed and requested not to perform any specific task under awake condition. In all the patients, CCEPs were recorded in the second week after returning the dosage of antiepileptic drugs. At least 2 trials of 30–50 responses each were averaged to confirm the reproducibility of responses. CCEPs were obtained off-line by averaging ECoGs time-locked to the stimulus onset. The time window (1,000 ms) and baseline (200 ms) was set as the same as those of SEPs. The method has been reported elsewhere in detail [46,58].

### Definition of the Primary Sensory Cortex (SI) and Selection of CCEPs in the hand SI

In this study, we aimed at comparing HFAs overriding SEPs and CCEPs in the hand SI. The hand SI electrode was identified according to the largest cortical SEP component, N20. We confirmed the location of the hand SI electrodes on the postcentral gyrus anatomically by the 3D-MRI in all patients. The epileptic focus was away from the hand SI in all patients. No patients showed interictal epileptic spikes at the hand SI electrode.

We usually perform SPES to most of the implanted electrodes in order to investigate the cortico-cortical connections involved in seizure propagation and brain functions for clinical purposes. For this particular study, we selected the stimulus sites (electrode pairs) that produced large outstanding CCEP waveforms at the hand SI electrode in each patient. The subsequent time frequency analysis was performed for the ECoG data for each CCEP stimulus site. In other words, when the robust CCEP waveform was recorded in the hand SI from more than 1 stimulus site (e.g., 2 stimulus sites) in 1 patient, the time frequency analysis was performed for each stimulus site separately.

### Time Frequency Analysis

A time-frequency representation was built for each epoch of raw ECoG data recorded during SEP and CCEP recording by using the short-time Fourier Transform (STFT). The epoch duration was the same as that used for SEP and CCEP analyses, i.e., from 300 ms before to 700 ms after the stimulus onset. The analysis frequency range was 0–600 Hz. The Fourier Transform was performed on 25 data-point window (12.5 ms; frequency resolution 80 Hz) at each time-step. The step of the sliding window was set at 5 ms; in other words, 1 time bin was with width of 5 ms centered at 2.5 ms. A Hanning window was imposed on each window to attenuate edge effects.

With the current amplifier (EEG-1100, Nihon Kohden, Tokyo, Japan), the stimulus artifact lasts up to 3–4 ms from the stimulus onset. A preliminary CCEP latency analysis revealed that N1 peaked between 7–27 ms at the SI electrode. In order to differentiate the stimulus artifact from the N1 potential for the STFT anlaysis, we selected the short window size of 25 points (12.5 ms) and sacrificed the frequency resolution to 80 Hz. When we analyze the high frequency activities, it is important to distinguish the evoked response that is time-locked and phase-locked to the stimulation from the induced response that is time-locked, but not phase-locked. Induced (non-phase-locked) responses are analyzed by subtracting event-related potential from the raw ECoG signal in each individual trial to minimize the contribution of evoked (phase-locked) responses [59]. In this study, our aim was to analyze neuronal activities induced by external stimulation upon individual trial basis. Therefore, in contrast to the most of the previous somatosensory evoked HFA studies, we adopted induced HFA responses for SEPs in the same condition as those of CCEPs.

After the STFT, we averaged the power spectrum across all the epochs. The logarithmic power spectrum (base 10) was computed for the given frequency range and window. The baseline for computation was set to the same as that for averaging SEP and CCEP: from 300 ms to 100 ms before the stimulus onset. We refer to the stimulus-locked induced HFA for SEP data as “HFA_SEP_” and that for CCEP data as “HFA_CCEP_” herein for clarity. For HFA_CCEP_ analysis, we focused on the induced HFA overriding the early N1 potential (HFA_CCEP(N1)_) and the following N2 potential (HFA_CCEP(N2)_). Since the N1 is regarded as the first volley reaching the target cortex [49,51,60], HFA overriding N20 (HFA_SEP(N20)_) was analyzed as an N1 counterpart. HFA overriding N80 (HFA_SEP(N80)_), a late cortical component subsequent to the early cortical component, was also analyzed since the peak latency was closest to that of N2. As for HFAs on the early potentials (HFA_SEP(N20)_, HFA_CCEP(N1)_), the power changes were calculated for 1 time bin that included the peak of SEP N20 or CCEP N1. We carefully selected a bin after 15 ms for evaluating HFA_CCEP(N1)_ and HFA_SEP(N20)_ so that the 25 data-point (12.5 ms) window did not overlap with the stimulus artifact in all patients. CCEP N1 peaked, however, before 12.5 ms in 2 CCEP responses, and we selected the bin at 15 ms instead in order to avoid stimulus artifact on these rare occasions. We selected 4 bins (mean of 20 ms bin at and around the peak) for calculating the power spectra of CCEP N2 and SEP N80. We confirmed the validity of the usage of the bin after 15 ms for evaluating HFA_CCEP(N1)_ and HFA_SEP(N20)_, namely, that the 25 data-point (12.5 ms) window did not overlap with the stimulus artifact, by performing an additional stimulus artifact analysis using the dead pig brain (see S1 Text and S1 Fig).

**Fig 1:**
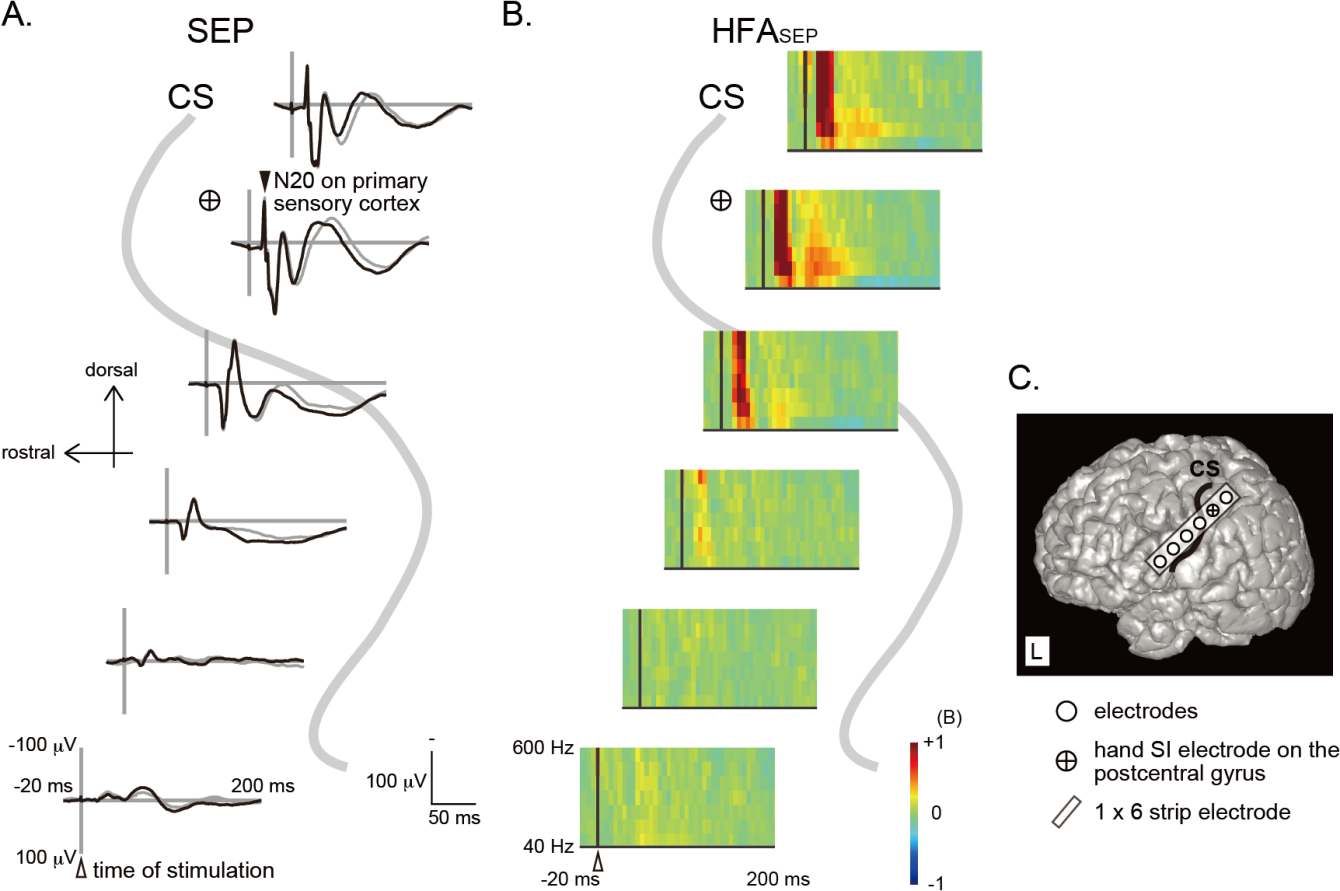
SEPs and HFAs_SEP_ recorded from the peri-rolandic area and 3D-MRI (patient 1, left hemisphere). A: SEPs to right median nerve stimulation are plotted with subaverages (black and grey waveforms) across the CS identified on 3D-MRI (in a representative case). The vertical line corresponds to the time of median nerve stimulation (a white arrowhead). N20 component showing the maximum amplitude is identified on the primary somatosensory cortex (SI) (a black arrowhead). B: Time-frequency representation of SEP to right median nerve stimulation (HFA_**SEP**_) by using the short-time Fourier Transform is shown across the CS. The frequency range is from 40 to 600 Hz. The vertical line corresponds to the time of median nerve stimulation (a white arrowhead). The averaged logarithmic power spectrum in reference to the baseline is calculated. Increase of power is indicated in red and decrease in blue. C: On 3D-MRI, subdural electrodes are plotted as white circles. A hand SI electrode is plotted as a white circle with a cross. Only electrodes at and around the hand SI and stimulus electrodes are shown in the figure. Since most of the induced high frequency activities were within 200 ms from the stimulus onset, we displayed the STFT results across the whole time points and frequencies in 3 dimensions (time, frequency, and power value) in a time window of 220 ms (from 20 ms before to 200 ms after the stimulus onset). SEP, somatosensory evoked potential; HFA, high frequency activity; CS, central sulcus.

**Fig 2:**
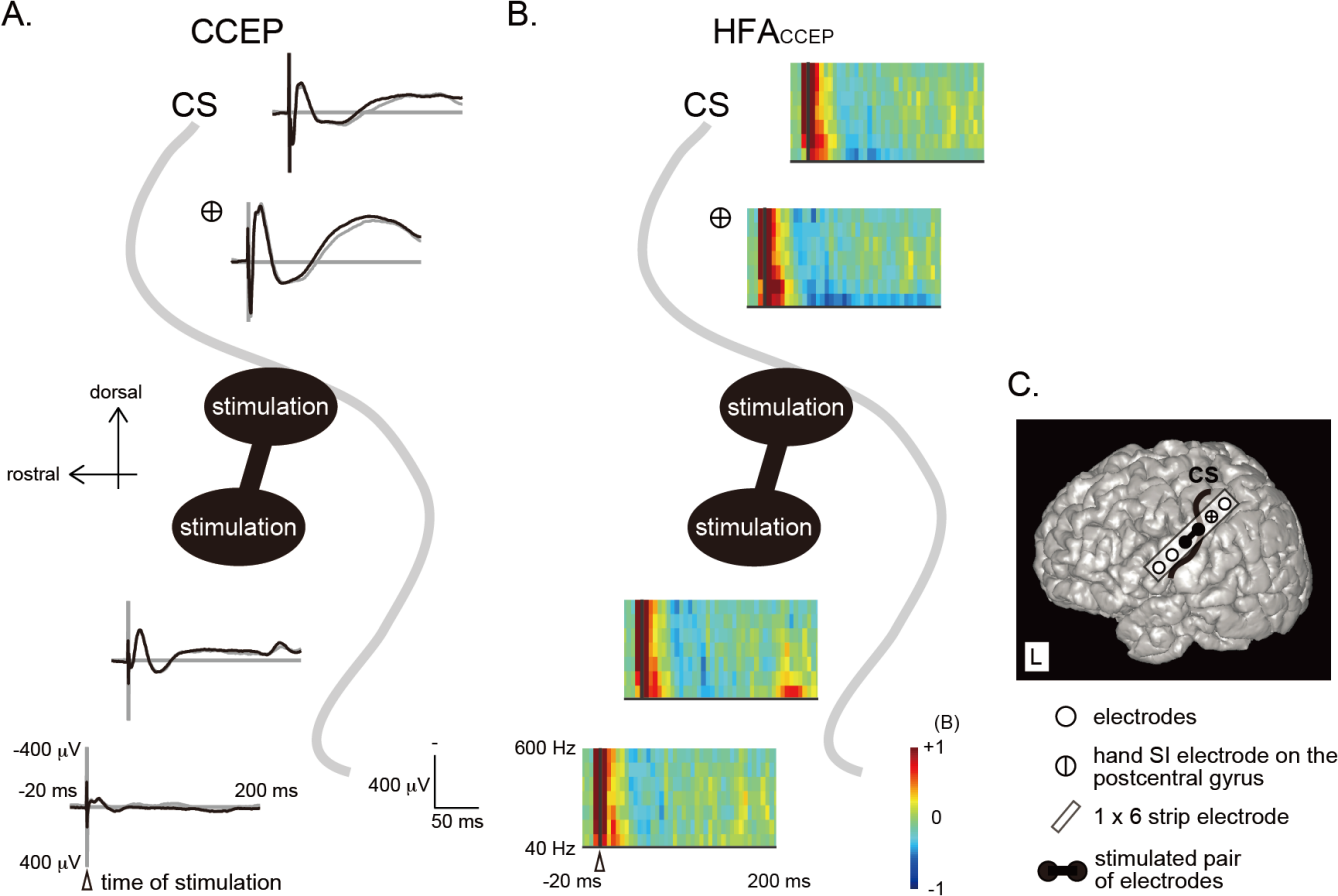
CCEPs and HFAs_CCEP_ recorded from the peri-rolandic area and 3D-MRI (patient 1, left hemisphere). A: Single pulse stimulation was applied to the electrodes on the precentral gyrus and CCEPs were recorded time-locked to the stimuli (in a representative case). Two subaverages (black and grey waveforms) are shown. The vertical line corresponds to the time of single pulse stimulation (white arrowhead). B: Time-frequency representation of CCEP (HFA_**CCEP**_) by using the short-time Fourier Transform. C: Electrodes on 3D-MRI. CCEP = cortico-cortical evoked potential. Other conventions are the same as for Fig 1.

**Fig 3:**
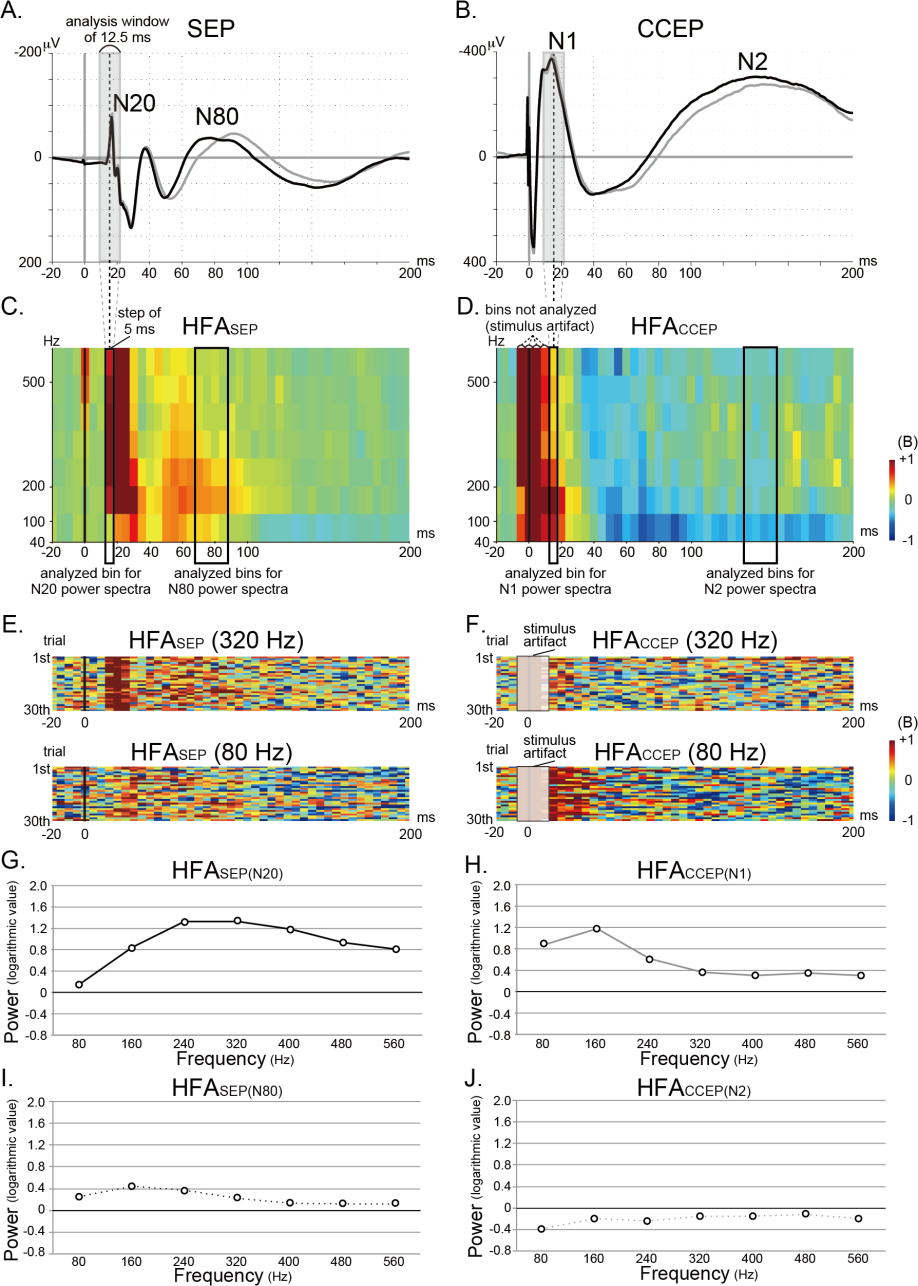
SEP, CCEP, HFA_SEP_, and HFA_CCEP_ at SI (patient 1, left hemisphere). A-D: SEP (A), CCEP (B), HFA_**SEP**_ (C), and HFA_**CCEP**_ (D) recorded from the same hand SI electrode are shown in a representative case. The STFT was performed by using the short analysis-window of 25 points (12.5 ms) in order to differentiate the stimulus artifact from the CCEP N1 potential. Since the sliding window is set at 5 ms, each time bin (5ms-width) displays the STFT results of the 12.5 ms analysis-window. For example, the 5 ms-time bin centered at 15 ms (highlighted by a black rectangle in C and D) corresponds to the results of 12.5 ms analysis-window (from 9 ms to 21.5 ms, centered at 15 ms; see shaded gray rectangle in A and B). The stimulus artifacts in CCEP last up to 3–4 ms from the stimulus onset. Therefore, the bins centered at -5, 0, 5, and 10 ms potentially include the stimulus artifacts and they are not analyzed. Because we put the transistor-transistor logic (TTL) pulse from the electric stimulator into the DC input of the EEG machine, and offline triggered the stimulus onset using a certain threshold with a Matlab-script, the trigger timing could have jitter within the sampling point, namely, 0.5 ms. This jitter is reflected in the representative CCEP waveform (B). As for the induced activities, the 5 ms time bins centered at -5 and 0 ms, which correspond to the results of 12.5 ms window centered at -5 and 0 ms, could include the stimulus artifact (D). E, F: The row traces (30 trials) of HFA_**SEP**_ (E) and HFA_**CCEP**_ (F) for the frequency bands centered at 80 and 320 Hz are shown. G-J: The power changes of HFA_**SEP(N20)**_, HFA_**CCEP(N1)**_, HFA_**SEP(N80)**_, and HFA_**CCEP(N2)**_ in reference to the baseline activity for each frequency band (every 80 Hz, centered at 80, 160, 240, 320, 400, 480, and 560 Hz) are plotted (G, H, I, and J).

Since most of the aforementioned induced HFA activities were within 200 ms from the stimulus onset, we displayed the STFT results across the whole time points and frequencies in 3 dimensions (time, frequency, and power value) in a time window of 220 ms (from 20 ms before to 200 ms after the stimulus onset) in figures (Figs 1B, 2B, 3A and 3B).

**Fig 4:**
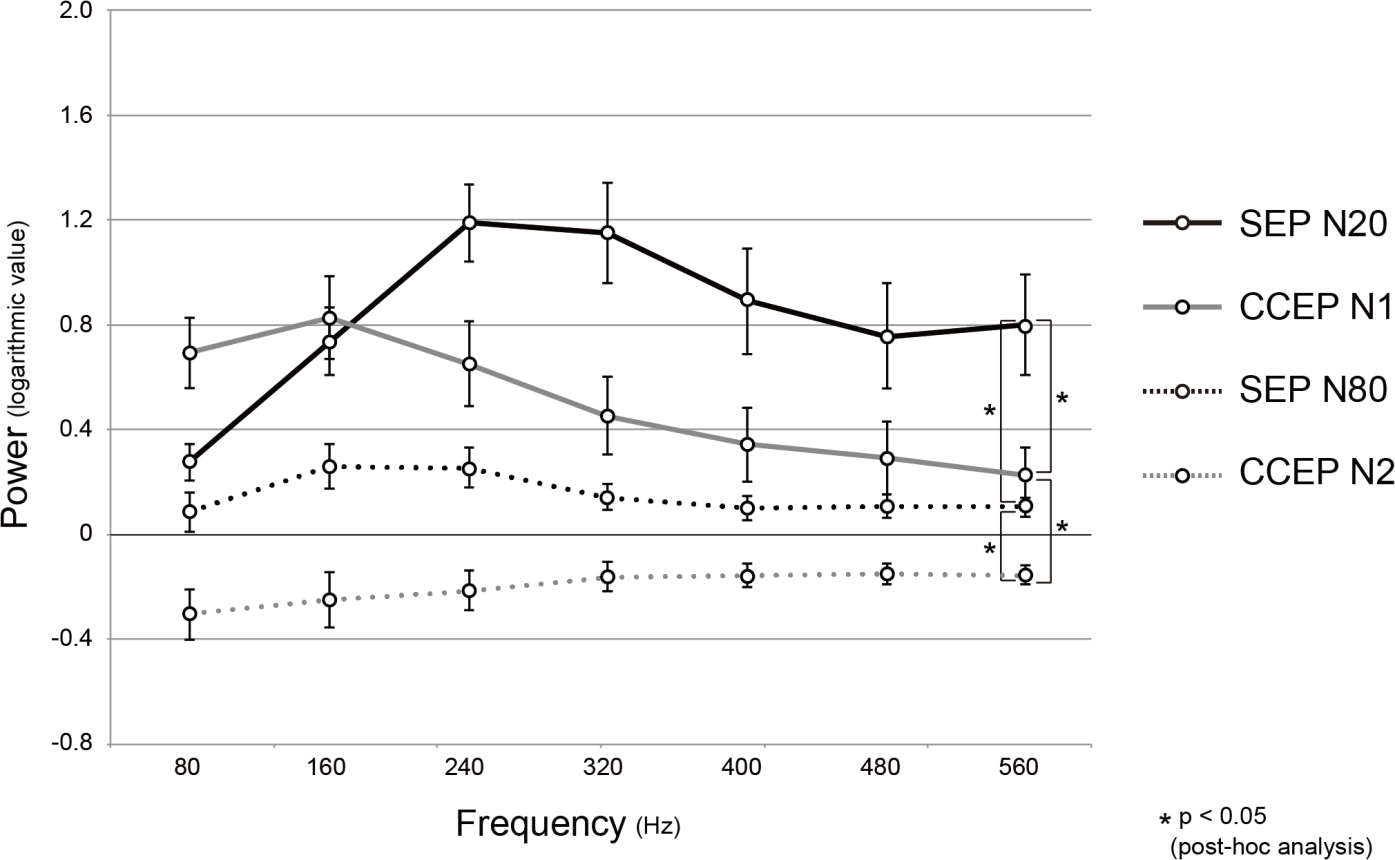
The distributions of logarithmic power values in reference to the baseline in each frequency band. As for the 4 groups, HFA_**SEP(N20)**_ (a black solid line), HFA_**CCEP(N1)**_ (a grey solid line), HFA_**SEP(N80)**_ (a black dashed line) and HFA_**CCEP(N2)**_ (a grey dashed line), all the power values of 7 hemispheres are averaged (mean ± SE). RM-ANOVA showed statistically significant interactions between the 4 groups. An asterisk indicates significant interaction between the 2 groups in the post-hoc analysis. Other conventions are the same as for Fig 3.

In order to compare the power trend across the frequency bands among HFA_SEP(N20)_, HFA_SEP(N80)_, HFA_CCEP(N1)_, and HFA_CCEP(N2)_, we also drew additional figures plotting the change of the logarithmic power spectra across the frequency bands at the timing of N1 and N2 of CCEP and N20 and N80 of SEP. In each patient, we analyzed the power values for frequency bands centered at 80, 160, 240, 320, 400, 480, and 560 Hz for the 4 groups (HFA_SEP(N20)_, HFA_SEP(N80)_, HFA_CCEP(N1)_, HFA_CCEP(N2)_). After the plot was made for each patient (see Fig 3G, 3H, 3I and 3J), all the power values from 7 hemispheres in 6 patients (N = 7 for HFA_SEP(N20)_ and HFA_SEP(N80)_, N = 16 for HFA_CCEP(N1)_ and HFA_CCEP(N2)_) were plotted in the same figure (see Fig 4).

### Statistical Analysis

The statistical analyses were performed by using the logarithmic power values of each SEP and CCEP responses (7 responses for HFA_SEP(N20)_ and HFA_SEP(N80)_, and 16 responses for HFA_CCEP(N1)_ and HFA_CCEP(N2)_). In this study we aimed to investigate whether there are differences of the distributions of logarithmic power spectra between the 4 groups. Repeated measures analysis of variance (RM-ANOVA) was adopted for the statistical investigation of the interactions with the power values in each frequency band as a within-group factor and with the aforementioned 4 groups [HFA_SEP(N20)_, HFA_SEP(N80)_, HFA_CCEP(N1)_, and HFA_CCEP(N2)_] as a between-group factor. As a post hoc analysis, we focused on the 4 comparisons between the groups: HFA_SEP(N20)_ and HFA_CCEP(N1)_, HFA_SEP(N80)_ and HFA_CCEP(N2)_, HFA_SEP(N20)_ and HFA_SEP(N80)_, and HFA_CCEP(N1)_ and HFA_CCEP(N2)_.

## Results

### Distribution of evoked responses and HFAs

Stimulation of the median nerve contralateral to the recording hemisphere elicited SEPs on the electrodes around the central sulcus (CS). As shown in the 3D-MRI of the representative case (patient 1L, Fig 1A), all the hand SI electrodes with the largest N20 were located on the postcentral gyrus. As for the power spectra, HFAs_SEP_ were recognized at and around the hand SI electrode (Fig 1B). The distribution of SEPs (N20 and N80) well corresponded to that of HFAs_SEP_ (Fig 1C) in all patients. Regarding distribution of CCEPs and HFAs_CCEP_, robust CCEP responses (N1 and N2) and induced HFAs_CCEP_ were recorded at the hand SI electrode by stimulating 16 stimulus sites (1–3 stimulus sites per patient: 1 site in patient 3, 2 sites in patient 1(L), 1(R), and 6, and 3 sites in patient 2, 4, and 5) (see patient 1L, Fig 2A and 2B for example).

### Comparison between HFA_SEP_ and HFA_CCEP_

Fig 3 shows an example of comparison between SEP/HFA_SEP_ and CCEP/HFA_CCEP_ at the hand SI electrode. HFA_SEP_ and HFA_CCEP_ were shown with the three dimensional scales along the time window of 220 ms (Fig 3C and 3D). The row traces of HFA_SEP_ and HFA_CCEP_ for the frequency bands centered at 80 and 320 Hz, at which the HFA_SEP_ and HFA_CCEP_ presented the different power trend, are shown (Fig 3E and 3F). The power changes at the N20/N1 and N80/N2 peaks were then plotted in the two dimensional scale to show the distribution of the power change across the frequency bands (Fig 3G, 3H, 3I and 3J). The frequency band showing the maximal power change differed between HFA_SEP(N20)_ and HFA_CCEP(N1)_, HFA_SEP(N20)_ and HFA_SEP(N80)_, HFA_CCEP(N1)_ and HFA_CCEP(N2)_, and HFA_SEP(N80)_ and HFA_CCEP(N2)_ in this representative case (patient 1L). In order to compare the distribution of the power change between HFA_SEP_ and HFA_CCEP_ across patients, the mean and standard error of the logarithmic power values of all the responses are plotted across the frequency band for HFA_SEP(N20)_, HFA_SEP(N80)_, HFA_CCEP(N1)_, and HFA_CCEP(N2)_ (Fig 4). It showed a tendency that over 200 Hz, the HFA_SEP(N20)_ power increased while HFA_CCEP(N1)_ power decreased. RM-ANOVA showed statistically significant interactions between the 4 groups (F(3,6) = 14.468, p < 0.05). A post-hoc analysis showed significant interactions between HFA_SEP(N20)_ and HFA_CCEP(N1)_ (F(1,6) = 19.409, p < 0.05), HFA_SEP(N20)_ and HFA_SEP(N80)_ (F(1,6) = 9.280, p < 0.05), HFA_CCEP(N1)_ and HFA_CCEP(N2)_ (F(1,6) = 22.494, p < 0.05), and HFA_SEP(N80)_ and HFA_CCEP(N2)_ (F(1,6) = 4.097, p < 0.05). In other words, the distributions of power values differed between the 2 early components (CCEP N1 vs. SEP N20), as well as the 2 late components (CCEP N2 vs. SEP N80), and also between the early and late components of SEPs or CCEPs (SEP N1 vs. N2, CCEP N1 vs. N2).

## Discussion

By applying SPES both to the peripheral nerve and directly to the cortex, we compared the HFAs overriding SEPs and CCEPs in the SI cortex. Stimulus-triggered HFAs behaved differently in terms of power distribution. As for the early components, HFA_SEP(N20)_ power increased more in the frequency band above 200 Hz while HFA_CCEP(N1)_ in the band below 200 Hz. As for the late components, statistically significant difference was also observed for the distributions of power spectra between HFA_SEP(N80)_ and HFA_CCEP(N2)_. Both HFA_SEP_ and HFA_CCEP_ showed different power distributions between early and late components. The present findings indicated that the human sensory cortex could produce different HFA profiles upon the different modes of input.

### Stimulus induced early HFAs

As for the gamma activities recorded with macroelectrodes, Ray et al., by using a macaque monkey model, showed that the high gamma activity sensitively increased both in association with neuronal synchrony and firing rate [16]. In this regard, we think the HFA or ‘broadband’ gamma profiles were determined by neuronal synchrony and firing rate induced by the 2 modes of input in the present study. The HFAs were overriding or associated with either SEPs or CCEPs. SEPs are evoked through vertical, ascending projection fibers from the thalamus to the SI cortex, and CCEPs are evoked through horizontal association or commissural fibers based on the previous reports [51,60]. It is, therefore, plausible to ascribe the different gamma profiles between the early HFA components, HFA_SEP(N20)_ and HFA_CCEP(N1)_, to the different modes of impulse transmission through vertical projection vs. horizontal association/commissural fibers. The difference, then, could be explained by the different layer input from these 2 afferent fibers. Association and commissural fibers originate from the layer II and III and terminate mainly in the same layers elsewhere in the neocortex [61], while projection fibers from the thalamic relay (specific) nuclei, e.g. ventral posterolateral (VPL) in case of median nerve stimulation, terminate mainly in a rich arborization within the layer IV [62]. In this sense, it is indicated that different input modes played an important role in subsequent neuronal processing in terms of frequency tuning: neuronal synchrony in the high gamma range in case of horizontal transmission (HFA_CCEP(N1)_ <200 Hz) vs. above the high gamma range in case of vertical transmission (HFA_SEP(N20)_ >200 Hz). Several animal studies have indeed revealed that frequency tuning (alpha, beta and gamma) for neural information processing differs according to the cortical layers, while that of faster activities above the conventional gamma range has not been well elucidated [45,63–68]. Since SI has the most developed layer IV in the neocortex, the inputs to the layer IV from the specific nuclei of the thalamus might give rise to neuronal synchrony in the higher frequency range than those to the layer II/III. A possible key player of the synchronization above 200 Hz is at least the fast-spiking interneuron which could produce the high frequency activities ranging 300–500 Hz [69–70]. In summary, different frequency profiles between HFA_SEP(N20)_ and HFA_CCEP(N1)_ is most likely due to the different propagation modes (vertical vs. horizontal), namely, the different terminal layers (layer IV vs. II/III) where afferent inputs arrive within SI. The layer IV contains the fast-spiking interneurons more than the layer II/III in SI. We speculate that the difference of the distribution of the fast-spiking interneurons may partly determine the most prominent frequency band of somatosensory and cortico-cortical HFAs in SI.

Another possible explanation is the influence of temporal jitters. ‘Direct cortical response (DCR)’ in the vicinity of the site of direct cortical stimulation has been extensively studied in various species [71–73]. Simultaneous surface and intracellular recording in animals revealed that the first negative components of DCR reflect oligosynaptic events in the local cortical circuits [46,74–75]. This local jitter of synaptic activity at the site of stimulation and at the target cortex in CCEPs might create the lesser synchronization as compared with the relatively synchronized disynaptic activities observed in SEPs.

The HFA_CCEP(N1)_ has a frequency profile similar to HFA induced during various cognitive tasks (‘cognitive HFA’). Cognitive HFAs have been reported to show a broadband shape in the power spectrum, which is probably organized by multiunit activities and/or local field potentials [16–17] or summation of membrane potential oscillations with different center frequencies [76]. Taking account of the similar frequency profile, cortico-cortical connections is highly likely to represent common mode of neuronal transmissions for various brain functions although the interaction to the ‘centrencephalic’ area would also influence the brain functions.

### Stimulus induced late HFAs

The frequency profile was also different between the late components, HFA_SEP(N80)_ and HFA_CCEP(N2)_. Both SEP N80 and CCEP N2 have been considered to be late cortical components, although it is still not clear what they exactly reflect. One possible explanation for different frequency profiles in late HFAs is that the different stimulus types, a physiological stimulus through the peripheral nerve stimulation and a non-physiological stimulus directly applied to the cortex, could produce different intracortical neural processing in SI. In contrast to HFA_SEP(N80)_ that had increased power in reference to the baseline activity, HFA_CCEP(N2)_ showed a decline of the power value. This tendency is similar to the postspike depression seen in the spikes abnormally or non-physiologically produced by the epileptic focus [77–79]. It has been considered that this postspike depression reflects the decreased cortical excitability after paroxysmal depolarization shifts for epileptic spikes. In addition, by using an SPES technique similar to our CCEP method and recording multiunit activities, Alarcon et al. (2012) revealed that neural responses induced by SPES consist of brief synchronized burst firing and subsequent long suppression [80]. Therefore CCEP N2 could reflect an inhibitory process after the excitation reflected in N1 and HFA_CCEP(N1)_. This might be an essential compensatory function of the human cerebral cortex. On the other hand, Matsuzaki et al. (2013), in their SPES/CCEP study in children, recently reported the increased gamma activities at the time of the end of N2 response in the visual cortex [81]. The difference between our study (decreased power) and theirs (increased power) could be explained by the difference of the cytoarchitecture, the patients’ age and the maturity of brain, or the phase of CCEP N2 response (peak vs. descending slope) when HFAs were observed. In our study, the bins centered at 80 Hz showed a tendency of the relative power decrease compared with the bins centered at 160 Hz, especially HFAs for CCEP. This is explained by the possibility that the frequency range centered at 80 Hz is mostly controlled by the inhibitory interneurons.

### Clinical implications and limitations

Since this study was performed in patients with epilepsy or brain tumor, we should discuss whether we recorded pathological or physiological HFAs. In all patients, no epileptic HFAs or epileptic spikes were observed at the hand SI electrode. Together with the normal SEP configuration at and around the SI electrode, we considered the HFA_SEP_ and HFA_CCEP_ observed in the hand SI as physiological.

Pathological HFAs in the frequency range of ripple (100–200 Hz) and fast ripple (250- Hz) have recently attached considerable attention as a possible surrogate marker of epileptogenicity. By measuring the amplitude, CCEPs have been used for evaluating epileptogenicity or cortical excitability at and around the focus [55–56,82]. In this study we demonstrated the power increase (HFA_CCEP(N1)_) and decrease (HFA_CCEP(N2)_) were associated with CCEPs in the normally functioning SI. Besides the amplitude, the HFA correlates of CCEPs, namely HFA_CCEP(N1)_ and HFA_CCEP(N2)_, could also be clinically useful to evaluate the degree of abnormally enhanced cortical excitability and also surround inhibition at and around the epileptic focus, respectively.

There are several limitations in this study. First, the STFT analysis period was set after 15 ms in order to avoid the possible involvement of stimulus artifacts in the analysis. Therefore, some HFAs_CCEP_ at the time range of 0 to 10 ms, such as HFA_CCEP_ overriding CCEP first volley or P1, which is the very first response reported in CCEP studies [60,83], could not be evaluated in this study. The relatively short analysis window (12.5 ms) and resultant broad frequency bin (80 Hz) did not allow us to analyze lower frequencies such as the beta or low gamma (below 40 Hz) band in the present study. Viswanathan et al. (2007) and Ray et al. (2008) reported that high-gamma (60–90 or 60–200 Hz) power could be a neural correlate of synchronized output of the cortex, while low-gamma (25–60 or 40–80 Hz) power could be a correlate of synchronized input to the cortex [16,84]. In this study, we mainly dealt with the activities of high-gamma range that reflects spike synchronization due to the 80 Hz frequency resolution. Therefore, our study suggests that the different propagation modes caused the frequency difference (HFAs_SEP_ vs. HFAs_CCEP_) within high-gamma range. Although the difference of low-gamma activities might have some influence on our results, synchronized input to the cortex would affect the neural correlate of synchronized output of the cortex. Second, it has been reported that human somatosensory evoked HFAs consist of 2 phases: early HFAs seen before the N20 peak and late HFAs after the peak. Possible generators of early HFAs are action potentials of the thalamocortical fibers at the time when they arrive at the area 3b (and area 1), and those of late HFAs are cortical fast inhibitory postosynaptic potentials or fast spiking interneurons in SI. In this study, we could not differentiate early and late HFAs due to the analysis window. Therefore, the difference of HFAs overriding SEP and CCEP might also reflect that of the presynaptic HFAs. Even in this case, the difference of the propagation modes and terminal layers between SEPs and CCEPs would be a key factor. Third, antiepileptic drugs might influence the occurrence of HFAs. The occurrence of pathological HFAs was reported to increase after reduction of antiepileptic drugs and decrease after induction of propofol [38,85]. Somatosensory evoked HFAs were reported to decrease both in frequency and amplitude during propofol administration [86–87]. In our study, although the SEP and CCEP recordings were not performed on the same day in each patient, the amount of antiepileptic medication was almost the same between the 2 recordings. We, therefore, think that antiepileptic drugs hardly influenced the comparison between HFA_SEP_ and HFA_CCEP_. Fourth, we investigated the SI cortex since we could record and compare stimulus induced HFAs in response to both physiological (median nerve stimulation) vertical and non-physiological (direct electrical cortical stimulation) horizontal inputs. It is technically difficult to perform a similar study in the association cortex, but we assume the findings in SI could be applicable to the neocortex in general. Lastly, the sampling rate (2,000 Hz) and filter setting (0.016–600 Hz) prevented us to evaluate the HFA over 600 Hz. Our objective was to compare behaviors of the HFA involved in physiological and epileptic broadband gamma range, and we successfully demonstrated different behaviors in this frequency range for HFAs_CCEP_ and HFAs_SEP_. Future studies sacrificing the number of electrodes to increase the sampling rate would warrant investigation of higher HFA which was out of scope in the present study.

## Supporting Information

S1 Text Additional Data for Time Frequency Analysis. (DOC)

S1 Fig CCEPs and HFAs_CCEP_ in an additional study to validate the influence of the stimulus artifacts. A: A configuration of grid electrodes placed on each hemisphere of a dead brain of a pig. Shaded circles indicate 2 stimulated pairs of electrodes. B: CCEPs recorded time-locked to the single pulse stimulation applied to the electrodes labeled as “1” in S1A Fig. Two subaverages (black and grey waveforms) are shown. We displayed in a time window of 220 ms (from 20 ms before to 200 ms after the stimulus onset) in the same way as the original Figs 1–3. The vertical line corresponds to the time of single pulse stimulation (white arrowhead). C: A CCEP response recorded from the electrode adjacent to the stimulus sites (dotted square). D: Time-frequency representation of CCEP (HFA_CCEP_) by using the short-time Fourier Transform. The frequency range is from 40 to 600 Hz. The vertical line corresponds to the time of single pulse stimulation (a white arrowhead). The averaged logarithmic power spectrum in reference to the baseline is calculated. Increase of power is indicated in red and decrease in blue. E: An HFA_CCEP_ recorded from the same electrode as S1C Fig. In this particular example, the stimulus artifact, indicated in dark red suggestive of extraordinary power increase, affected the time bin centered at 5 ms and did not affect the bins centered at 10 ms and 15 ms. In case of stimulation of another pair of electrodes (labeled as “2” in S1A Fig), the stimulus artifact never influenced to the bin centered at 15 ms.
(TIF)
