# Supplementary material for: Different Mode of Afferents Determines the Frequency Range of High Frequency Activities in the Human Brain: Direct Electrocorticographic Comparison between Peripheral Nerve and Direct Cortical Stimulation
Source: PLoS One. 2015 Jun 18;10(6):e0130461. doi: 10.1371/journal.pone.0130461 (PMC4472671; doi:10.1371/journal.pone.0130461)
Supplement: S1 Text — (DOC) [file pone.0130461.s001.doc]

**Additional Data for Time Frequency Analysis**

In order to validate the influence of the electrical stimulus artifacts on the evoked potentials (CCEPs) and power spectrum (HFAs_CCEP_), we performed an additional animal experiment. We prepared a dead brain of a pig that was provided for the edible purpose. The brain for this experiment was smaller than that of human, and taken out from the body. Exactly the same grid electrodes and equipment (the EEG machine and stimulator) as those in the main study were used for this experiment. The electrodes were placed on each hemisphere. ECoGs were recorded under the same condition as those recorded for acquisition of CCEPs and analyzed off-line. The recordings were referenced to an electrode on the dorsal surface of the cerebellum. Stimulus parameters were the same as for the main study except for the stimulus intensity (15 mA) in 1 stimulus site that was set higher in order to explore the largest stimulus artifacts. CCEPs and time frequency representations were built using ECoG responses to 2 trials of 30 stimuli by using the same parameters as for the main study. We stimulated the 2 pairs of electrodes to confirm the reproducibility. The impedance was kept below 20 kohm, which was almost same as that during the main study. At the electrodes adjacent to the stimulus sites, the stimulus artifact lasted up to 4 ms from the stimulus onset in the raw ECoGs and CCEPs. In the time frequency analysis, the stimulus artifacts affected the time bin centered at 5 or 10 ms but never influenced the bin centered at 15 ms (see S1 Fig. for results). This experiment firmly confirmed the validity of the usage of the bin after 15 ms for evaluating HFA_CCEP(N1)_ and HFA_SEP(N20)_, namely, that the 25 data-point (12.5 ms) window did not overlap with the stimulus artifact.
