# Supplementary figures and images for: Different Mode of Afferents Determines the Frequency Range of High Frequency Activities in the Human Brain: Direct Electrocorticographic Comparison between Peripheral Nerve and Direct Cortical Stimulation
Source: PLoS One. 2015 Jun 18;10(6):e0130461. doi: 10.1371/journal.pone.0130461 (PMC4472671; doi:10.1371/journal.pone.0130461)

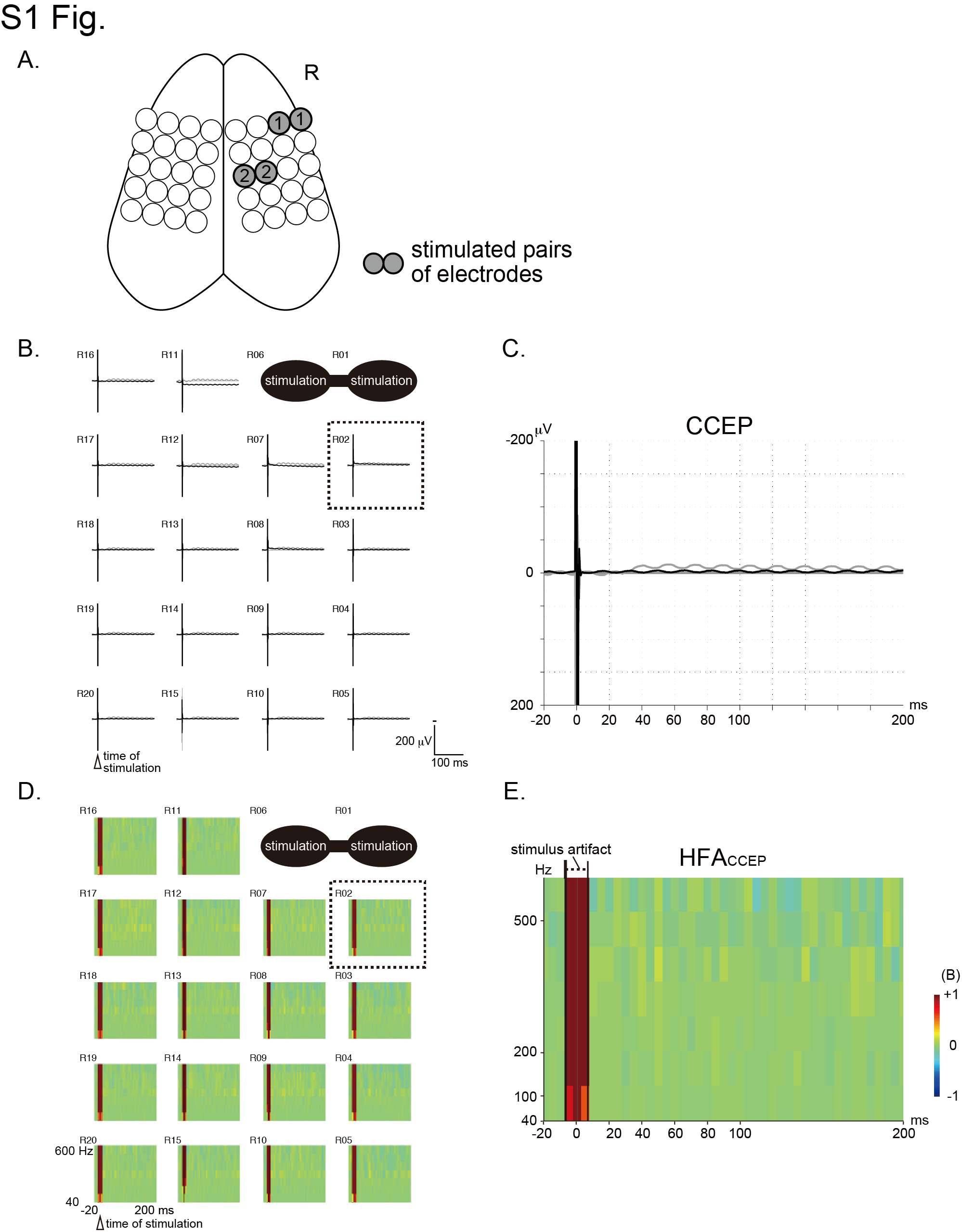

Supplement: S1 Fig — A: A configuration of grid electrodes placed on each hemisphere of a dead brain of a pig. Shaded circles indicate 2 stimulated pairs of electrodes. B: CCEPs recorded time-locked to the single pulse stimulation applied to the electrodes labeled as “1” in S1A Fig. Two subaverages (black and grey waveforms) are shown. We displayed in a time window of 220 ms (from 20 ms before to 200 ms after the stimulus onset) in the same way as the original Figs 1–3. The vertical line corresponds to the time of single pulse stimulation (white arrowhead). C: A CCEP response recorded from the electrode adjacent to the stimulus sites (dotted square). D: Time-frequency representation of CCEP (HFACCEP) by using the short-time Fourier Transform. The frequency range is from 40 to 600 Hz. The vertical line corresponds to the time of single pulse stimulation (a white arrowhead). The averaged logarithmic power spectrum in reference to the baseline is calculated. Increase of power is indicated in red and decrease in blue. E: An HFACCEP recorded from the same electrode as S1C Fig. In this particular example, the stimulus artifact, indicated in dark red suggestive of extraordinary power increase, affected the time bin centered at 5 ms and did not affect the bins centered at 10 ms and 15 ms. In case of stimulation of another pair of electrodes (labeled as “2” in S1A Fig), the stimulus artifact never influenced to the bin centered at 15 ms. (TIF) [file pone.0130461.s002.tif]
